# Chylopericardial Tamponade Accompanied by Chylothorax after Right Upper Lobectomy: A Rare but Fatal Postoperative Complication

**DOI:** 10.70352/scrj.cr.25-0275

**Published:** 2025-07-02

**Authors:** Koichi Fukumoto, Reo Kondo, Madoka Goto, Yasuhisa Ichikawa, Hideki Tsubouchi, Mika Uchiyama, Shoichi Mori

**Affiliations:** Department of Thoracic Surgery, Japanese Red Cross Aichi Medical Center Nagoya Daiichi Hospital, Nagoya, Aichi, Japan

**Keywords:** postoperative chylothorax, chylopericardial tamponade, right upper lobectomy

## Abstract

**INTRODUCTION:**

Postoperative chylothorax after thoracic surgery is relatively rare, and cardiac tamponade accompanied by the former is a scarce phenomenon. Although there have been scarce reports of such cases in cardiac surgery, reports on general thoracic surgery are exceedingly rare.

**CASE PRESENTATION:**

A 57-year-old male with suspected lung cancer underwent right upper lobectomy and selective mediastinal lymph node dissection. Postoperatively, he developed chylothorax that was unresponsive to conservative management, and thoracic duct ligation was planned for postoperative day 13. However, on postoperative day 12, he experienced cardiopulmonary arrest secondary to chylopericardial tamponade. Although cardiac rhythm was restored by pericardiocentesis, resuscitation required 80 minutes. The patient ultimately died of multiple organ failure on postoperative day 23.

**CONCLUSIONS:**

To our knowledge, this is the first report of postoperative death due to chylopericardial tamponade accompanied by postoperative chylothorax. Although this is a scarce complication, it can be fatal, and thoracic surgeons who perform pulmonary resection with mediastinal lymph node dissection should be aware of this phenomenon. In cases of postoperative chylothorax with concurrent pericardial effusion, the possibility of developing chylopericardial tamponade should be considered, and active consideration of pericardial drainage is recommended.

## INTRODUCTION

Despite its relative rarity, postoperative chylothorax after pulmonary resection for lung cancer is often managed conservatively, and it rarely leads to postoperative mortality.^1,2)^ Postoperative chylopericardium is a scarce but potentially fatal situation.^3)^ Herein, we report a case of chylopericardial tamponade accompanied by postoperative chylothorax after right upper lobectomy and selective mediastinal lymph node dissection for primary lung cancer, which culminated in cardiopulmonary arrest and death.

## CASE PRESENTATION

**Fig. 1:**
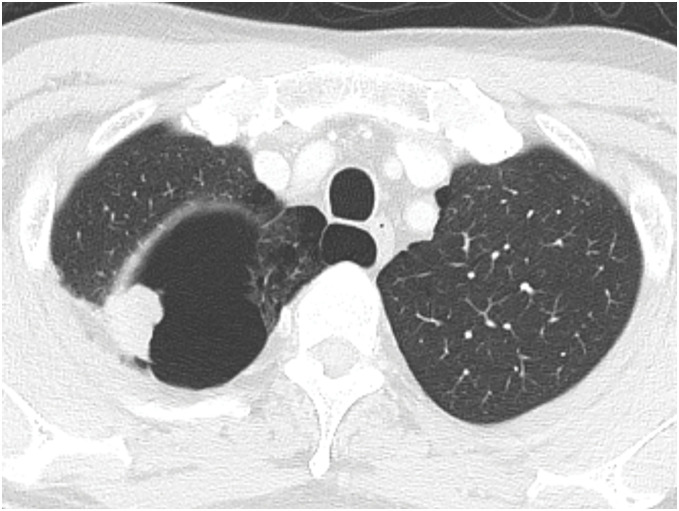
Preoperative chest CT image showing a solid nodule measuring 2.2 cm in the right upper lobe’s apical segment.

**Fig. 2:**
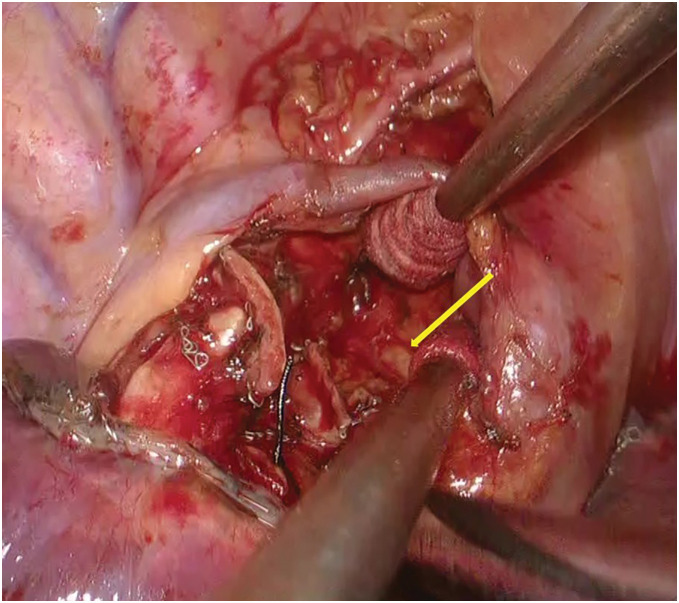
Intraoperative findings after right upper lobectomy and mediastinal lymph node dissection. A pericardial defect in the superior part of the pericardium near the azygos vein was incidentally created.

**Fig. 3:**
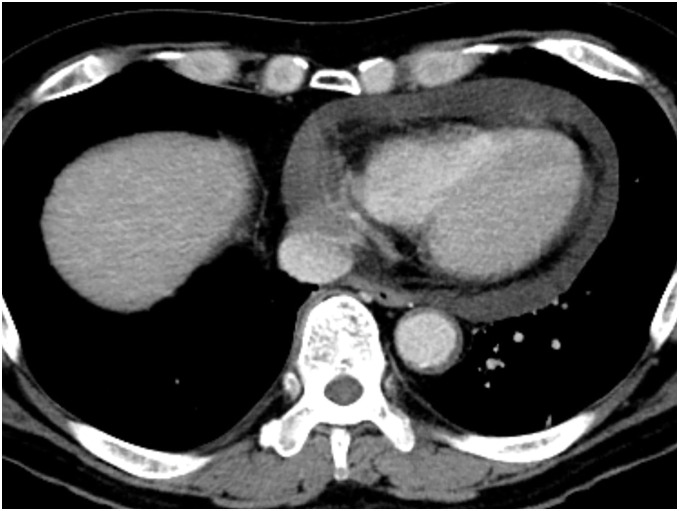
Enhanced CT image at the midnight of postoperative day 12 showing a moderate amount of pericardial effusion.

A 57-year-old male with suspected lung cancer was referred to our hospital for surgery. With a smoking history of 36 pack-years, he was on medication for hypertension and hyperlipidemia. An enhanced chest CT scan identified a 2.2-cm solid nodule in the right upper lobe, with no significant hilar and mediastinal lymph node enlargement (**Fig. 1**). The pulmonary nodule showed strong accumulation on fluorodeoxyglucose (FDG) positron emission tomography (PET)-CT (standardized uptake value [SUV] max: 16.7). Enhanced brain magnetic resonance imaging showed no signs of brain metastasis. Subsequently, the disease was staged as cT1cN0M0, stageIA3, which is suspected to be primary lung cancer, according to the 9th edition of the tumor-node-metastasis (TNM) lung cancer staging system. After an intraoperative frozen section procedure, the patient was diagnosed with non-small cell lung cancer and underwent right upper lobectomy with mediastinal lymph node dissection (ND2a-1) using multi-port video-assisted thoracoscopic surgery. At the point of mediastinal lymph node (station #4R) dissection, a tiny hole was made in the pericardial sac. Reconstruction was not performed as the hole measured approximately 5 mm in diameter (**Fig. 2**). A permanent histological examination of the operative specimen revealed a solid adenocarcinoma measuring 1.6 cm in total and invasive size, with no lymph node metastasis. The tumor was finally staged as pT1bN0M0, stage IA2. After the patient started postoperative oral intake, the chest drainage fluid became creamy on the 2nd postoperative day. The condition was diagnosed as postoperative chylothorax, as the drainage fluid’s triglyceride level was elevated (676 mg/dL). The patient’s oral intake was converted to a fat-restricted diet and injectable subcutaneous octreotide was initiated. On postoperative day 5, oral intake was discontinued and intravenous total parenteral nutrition via a peripherally inserted central catheter was initiated, as the amount of chest drainage fluid continued to be 700–800 mL per day. Despite discontinuation of the patient’s oral intake, the amount of chest drainage fluid did not decrease. Thoracic duct ligation under general anesthesia was scheduled for postoperative day 13. At midnight on postoperative day 12, the patient complained of sudden-onset severe back pain when he made an effort to defecate, which suggested aortic dissection, pulmonary embolism, or a urinary tract stone attack, among other possibilities. Emergent enhanced chest and abdominal CT showed no sign of the above-mentioned suspected diseases. Although a moderate amount of pericardial effusion was observed (**Fig. 3**), the patient was placed on an electrocardiogram monitor and followed up as the back pain dramatically disappeared during the CT scan without analgesic agents. In the early dawn of postoperative day 13, about 5 hours after the sudden onset of back pain, the patient suffered cardiopulmonary arrest (CPA) just after reporting chest discomfort. Despite the prompt cardiopulmonary resuscitation procedures, such as cardiac massage, artificial respiration with endotracheal intubation, and intravenous adrenaline administration, his heartbeat did not resume. Since the recent CT scan revealed moderate pericardial effusion (**Fig. 3**), pericardiocentesis was performed, during which 260 mL of yellow pericardial fluid was drained. The pericardial effusion’s triglyceride level was slightly elevated (115 mg/dL). The patient’s heartbeat resumed 14 minutes after pericardiocentesis; however, it took 80 minutes for the heartbeat to resume after CPA. Unfortunately, spontaneous breathing did not resume thereafter, and thoracic duct ligation was postponed. Electroencephalography on postoperative days 14 and 18 and a head CT scan on postoperative day 15 determined that the patient was nearly brain dead. He died of multiple organ failure on postoperative day 23. After the patient's death, a pathological autopsy was proposed to the family for a detailed study of the cause of death, but since consent was not obtained, no autopsy was performed.

## DISCUSSION

The incidence of postoperative chylothorax following anatomical resections and mediastinal lymph node dissection for lung cancer is reportedly 1.4%–4%. However, in most cases, the condition can be managed conservatively, and it rarely results in postoperative mortality.^1,2)^ In general thoracic surgery, postoperative chylopericardial tamponade accompanied by postoperative chylothorax is a rarity. To our knowledge, in the English literature, there have been only 2 case reports. Zhang et al. reported a case of postoperative chylothorax and chylopericardial tamponade following right lower lobectomy and lymphadenectomy.^3)^ This case is similar to ours, in terms of chylopericardium after lobectomy and lymphadenectomy in the right upper zone (Station #4R). Tam et al. reported a case of chylopericardial tamponade after posterior mediastinal mass resection.^4)^ In both cases, pericardiocentesis was the preferred treatment option, and the patient recovered from this fatal postoperative complication. To our knowledge, our case is the first to result in postoperative mortality in general thoracic surgery.

The mechanism of chylopericardium following postoperative chylothorax remains unclear; however, a rare anatomical variant may have caused the problem in our patient. In a cadaveric analysis, Riquet et al. reported that lymphatic channels in the pericardial sac were found to be connected from the heart to the thoracic duct in 8 out of 90 cases. They also found that all lymphatic channels from the left ventricle passed through the right lower paratracheal lymph node, while those from the right ventricle mainly passed through the left anterior mediastinal lymph node and were continuous with the thoracic duct.^5)^ The right superior mediastinal lymph node dissection is thought to cause intrapericardial lymphatic obstruction due to obstruction of lymphatic flow from the left ventricle, resulting in intrapericardial lymphatic obstruction caused by intrapericardial regurgitation due to lymphatic abnormalities such as valve regurgitation and increased intrapericardial pressure due to lymphatic obstruction. Reports of a similar condition after esophageal cancer surgery also support this hypothesis.^6)^ Similar to our case, in the case reported by Zhang et al., the pericardium was ruptured during mediastinal lymph node dissection.^3)^ How the ruptured pericardium contributed to the chylopericardium and cardiac tamponade is also unclear. We consider the possibility that adhesions may have occurred at some point at the site of the pericardial defect, obstructing lymphatic flow from within the pericardial sac and causing cardiac tamponade development. Another speculation by us is that the adhesions around the defective hole separated the thoracic cavity from the pericardial sac, causing lymphatic fluid to flow into the pericardial sac.

## CONCLUSIONS

We reported a case of cardiac tamponade accompanied by postoperative chylothorax after right upper lobectomy and selective mediastinal lymph node dissection for primary lung cancer. Although this is a scarce complication, it can be fatal, and thoracic surgeons who perform pulmonary resection with mediastinal lymph node dissection should be aware of this phenomenon. In cases of postoperative chylothorax with concurrent pericardial effusion, the possibility of developing chylopericardial tamponade should be considered, and active consideration of pericardial drainage is recommended.

## DECLARATIONS

### Funding

This study was funded by the Japanese Red Cross Aichi Medical Center Nagoya Daiichi Hospital Research Grant NFRCH 25-0007.

### Authors’ contributions

KF, HT, and YI performed the surgery, and KF, RK, MG, YI, HT, MU, and SM followed up the patient.

The manuscript was prepared by KF, under the supervision of SM.

The authors read and approved the final manuscript.

### Availability of data and materials

Data sharing is not applicable to this article, since datasets were neither generated nor analyzed for the case report.

### Ethics approval and consent to participate

This work does not require ethical considerations or approval. Informed consent to participate in this study was obtained from the patient.

### Consent for publication

Written informed consent for the publication of this case report was obtained from the patient.

### Competing interests

The authors have no conflicts of interest to disclose.

## References

[1] Bryant AS Minnich DJ Wei B The incidence and management of postoperative chylothorax after pulmonary resection and thoracic mediastinal lymph node dissection. Ann Thorac Surg 2014; 98: 232–5; discussion 235–7. 24811982 10.1016/j.athoracsur.2014.03.003

[2] Uchida S Suzuki K Hattori A Surgical intervention strategy for postoperative chylothorax after lung resection. Surg Today 2016; 46: 197–202. 26036222 10.1007/s00595-015-1183-6

[3] Zhang G Liu T Liang C. Chylothorax and chylopericardial tamponade following lobectomy and lymphadenectomy: a rare presentation. J Cardiothorac Surg 2023; 18: 25. 36647154 10.1186/s13019-023-02126-3 PMC9841673

[4] Tam CW Kumar SR. Chylopericardial tamponade after posterior mediastinal mass resection. J Cardiothorac Vasc Anesth 2020; 34: 2279–80. 32127276 10.1053/j.jvca.2019.12.034

[5] Riquet M Le Pimpec Barthes F Souilamas R Thoracic duct tributaries from intrathoracic organs. Ann Thorac Surg 2002; 73: 892–9; discussion 898–9. 11899197 10.1016/s0003-4975(01)03361-6

[6] Yang X Zhang J Sun P Chylopericardium following esophagectomy: a case report and systematic review. J Cardiothorac Surg 2024; 19: 50. 38310296 10.1186/s13019-024-02536-x PMC10838423

